# Hospitalisation for rotavirus gastroenteritis in the paediatric population in the Veneto Region, Italy

**DOI:** 10.1186/1471-2458-10-636

**Published:** 2010-10-22

**Authors:** Mario Saia, Aurore Giliberti, Giampietro Callegaro, Tatjana Baldovin, Marta Cecilia Busana, Francesco Pietrobon, Chiara Bertoncello, Vincenzo Baldo

**Affiliations:** 1 Hospital Services, the Veneto Region, Rio Novo, Italy; 2 Department of Environmental Medicine and Public Health, Institute of Hygiene, University of Padua, Italy; 3 Medical Directorate, Asolo Local Health Unit, Italy; 4 Regional Health Services Directorate, the Veneto Region, Rio Novo, Italy

## Abstract

**Background:**

This study evaluates the epidemiological impact of RVGE hospitalisation in the Veneto Region during the period spanning from 2000-2007 along with the associated costs. The analysis was conducted in an area where rotavirus vaccination is not included into immunization programmes and is an attempt to assess the potential benefits of such introduction.

**Methods:**

To update the estimates of acute RVGE hospitalisation rates in children ≤5 years in the Veneto Region, we conducted an 8 year retrospective observational population-based analysis (2000-2007).

**Results:**

Over the study period, a total of 4,119 admissions for RVGE were reported, with a mean hospital stay of 3.5 days. The population-based hospitalisation RVGE incidence rate was 195.8 per 100,000 children aged ≤5 years (lower than other European countries).

**Conclusions:**

RVGE is an important cause of paediatric hospitalisation in the Veneto Region. The data reaffirm the substantial burden of rotavirus hospitalisations in children and the potential health benefits of the vaccination as well as the possibility of adding rotavirus vaccination to the current schedule.

## Background

Rotavirus is the main cause of severe acute gastroenteritis (AGE) among children ≤5 years [1,2]. Improvements in sanitation and hygiene have not changed the global incidence of this disease, pointing to vaccination as the most effective way to achieve disease control [3,4]. Worldwide, the incidence of rotavirus gastroenteritis (RVGE) is similar both in developed and developing countries [2,5].

In industrialised countries, where access to health care is generally good, mortality due to RVGE is very low [6,7]. Nevertheless, the burden of disease remains considerable, being a major cause of hospitalization among children. In these countries, a model developed by the European Centre for Disease Prevention and Control estimates an overall rotavirus hospitalisation rate of 445 per 100,000 children younger than 5 years of age [3]. 

Globally, rotavirus is more common in the cooler months, but seasonal peaks can vary broadly and may occur from autumn to spring [8,9]. Rotavirus is primarily transmitted by fecal-oral route and is highly contagious. Almost all children are infected at least once before the age of 5 [3]. Its typical symptoms vary from a mild illness with self-limiting watery diarrhoea to severe diarrhoea accompanied by vomiting and fever, with risks of dehydration [2].

Gastrointestinal infections in children have a significant impact on the families and on society. They result in increased medical expenditures, lost productivity and childcare [10].

Vaccination is considered the most effective public health strategy to prevent rotavirus infection and reduce disease burden, so that the World Health Organisation strongly recommends inclusion of rotavirus vaccines into national immunisation programs in the regions where vaccine efficacy data suggest a significant public health impact and where appropriate infrastructure and finance mechanism**s **are available [11].

This study evaluates the epidemiological impact of RVGE hospitalisation in the Veneto Region during the period spanning from 2000-2007 along with the associated cost. The analysis was conducted in an area where rotavirus vaccination is not included into immunization programmes and is an attempt to assess the potential benefits of such introduction.

## Methods

To estimate the annual rates of RVGE associated hospitalization a retrospective population-based study was conducted analysing data collected from the hospital discharge database (HDD) of the Veneto Region (North East Italy), covering the 100% of discharges from all hospitals. The data were acquired in electronic form and were anonymous. The population used as denominator comes from the 2001 census data (National Institute of Statistics) and for the remaining years from the Office for Regional Statistics.

Cases of RVGE were identified using the International Classification of Diseases, nine revision, Clinical Modification (ICD-9-CM). Acute gastroenteritis discharges were identified by the codes ICD-9-CM 001 to 009, whereas RVGE discharges were identified by the code 008.61, eliminating secondary diagnosis so as to exclude nosocomial cases [12]. AGE cases of unspecified etiology were identified by code 009.1. The hospitalisation incidence due to rotavirus gastroenteritis and the mean hospital stay were calculated per 100,000 population. Mortality refers to inpatient mortality and does not consider the specific death cause.

In order to correlate the rate of hospitalization with the seasonal pattern the mean temperature by month is reported. Temperature data were supplied by the Regional Agency for environmental prevention and protection and derived from 14 monitoring stations located in different areas of the Veneto Region: 12 on plain (<300 m) and 2 on hill (300-600 m).

The direct costs associated to RVGE hospitalisations were estimated through DRG (Diagnosis Related Group) reimbursement rates. Rotavirus gastroenteritis has been referred to the DRG code 184 (esophagitis, gastroenteritis, and miscellaneous digestive disorders, age < 18) by considering only the first diagnosis. According to the Veneto regional DRG reimbursement system (HCFA-DRG, 24^th ^edition) the estimated cost of each RVGE hospital admission is 1,262 €.

The quantitative variables are expressed as the mean and the standard deviation (SD). The Odds Ratio (OR) was used to analyse variables such as age, sex and hospital stay, and the Chi square test for trend to analyse the seasonal pattern. A *P *value *<*0.05 was considered statistically significant.

The Hospital Services of the Veneto Region collect HDD. The data contained is recorded with the patient's consent and can be used as aggregated data for scientific studies without further authorizations. Furthermore the study complies with the Declaration of Helsinki and with the Italian Law Decree n. 196/2003 to protect personal data.

## Results

Over the 8-year study period, a total of 26,202 hospital discharges due to infectious gastroenteritis were registered (67.1 cases per 100,000 population). The mean hospital stay was 4.7 ± 5.7 days.

Of these hospitalisations, 14,201 (54.2%) occurred among children aged ≤5 years. Hospital rate among children was significantly higher than that in other age groups (OR = 20.8; [95% confidence interval: 20.30, 21.33]; p < 0.05) and the mean hospital stay halved (3.3 ± 2.4 days vs. 6.5 ± 5.7 days; p < 0.05).

Excluding AGE cases of unspecified etiology (37.8%), a total of 28.6% of all discharges due to AGE of specified etiology in children aged ≤5 years were attributable to RVGE.

The proportion of hospital discharges due to rotavirus gastroenteritis in different age groups was examined in order to assess age-specific rotavirus disease burden (Table 1). The majority of cases were reported among children ≤5 years, in particular infants ≤1 year which registered a significantly higher hospital rate and mean hospital stay than the other age groups (p < 0.05). During the study period 1 death occurred in a 2-year-old male.

**Table 1:** Hospital discharges attributable to rotavirus gastroenteritis (RVGE) by age groups

Age group	Number of hospitalisations due to RVGE (%)	Mean stay (SD)	RVGE hospital rate per 100,000
0-5 years	4,119 (91%)	3.5 (3.5)	195.8
*< 1 year*	969 (21%)	3.9 (3.3)	271.1
*1-5 years*	3,150 (70%)	3.4 (3.2)	180.4
Other ages	422 (9%)	3.3 (3.1)	1.1

Total	4,541 (100%)	3.5 (3.7)	11.6

SD: Standard deviation.

Table 2 shows the number of RVGE discharges, the mean hospital stay and hospital rate per 100,000 among children aged ≤5 years: minimal variations of the mean hospital stay were accounted against a significant hospital rate reduction (Chi-square for trend = 116.1; p < 0.05).

**Table 2:** Hospital discharges by rotavirus gastroenteritis (RVGE) among children ≤5 years from year 2000 to 2007

Year	Number of hospitalisations due to RVGE	Mean stay (SD)	RVGE hospital rate per 100,000	Proportion of hospitalisations due to RVGE in 0-5 years
2000	455	3.6 (2.9)	182.4	89%
2001	731	3.7 (3.1)	291.3	91%
2002	623	3.6 (3.2)	243.8	90%
2003	519	3.5 (3.1)	199.4	92%
2004	399	3.4 (3.4)	149.7	90%
2005	633	3.4 (3.3)	234	93%
2006	430	3.4 (2.7)	156.2	90%
2007	329	3.6 (3.4)	119.5	90%

**Total**	**4,119**	**3.5 (3.5)**	**195.8**	**91%**

SD: Standard deviation.

**Figure 1:**
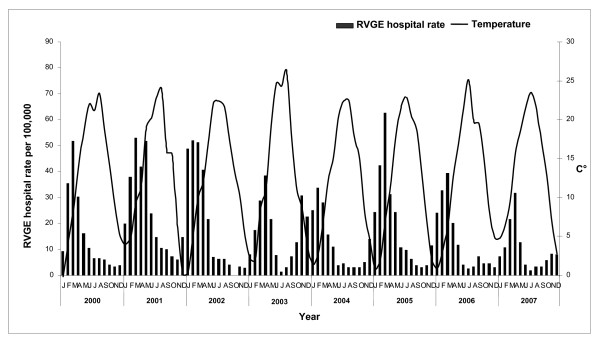
**Rotavirus hospitalisation rates of children ≤5 years and The mean monthly temperatures in the Veneto Region**.

Most RVGE cases occurred during the rotavirus season; infections peaked in winter and spring and were lower in summer (Figure 1), with 53.9% of discharges occurring in February-April and 21.4% in March.

The incidence of RVGE admissions in the Veneto Region among children aged ≤5 years was 195.8 per 100,000, ranking as the first cause of acute gastroenteritis hospitalisation for this age group. The mean hospital stay is equal to 3.5 days.

Over the 8-year study period, the economic impact of RVGE varied between 460,630 € and 1,009,600 €. Overall the cost of hospital admission was 5,730,742 € and the 91% of these were used for children ≤5 years.

## Discussion

The incidence of rotavirus infection in Italy seems substantially underestimated. As in most European countries detection of rotavirus is not notifiable so disease estimates are based on hospital discharge data [13]. A recent European model estimated about 10,000 hospitalisations, 80,000 physician visits, 320,000 episodes and 11 deaths occurring each year in Italy [5].

Our RVGE hospitalisation updated assessment confirms that rotavirus is a major cause of AGE among children aged ≤5 years, accounting 91% of RVGE cases.

The overall rate of hospitalisation for RVGE in our region among children aged ≤5 years is lower than the European mean (198 per 100,000 versus 300 per 100,000) and comparable with analogous studies conducted in other Italian regions [13-15]. However, the comparison with studies using national HDD should be made with caution because the timeframe, the at-risk population and the geographical districts do not overlap.

The mean hospital stay, equal to 3.5 days, is shorter than the European mean (4.8 days) but lies within the European range (2-9.5 days) [16]. Rotavirus gastroenteritis showed a considerable burden of hospital admissions and a clear seasonal distribution, with peak incidence from winter to spring as highlighted in other European countries [17,18].

Although most of rotavirus infections are successfully treated in a primary care setting, hospitalisation-related costs account for 80% of the budget destined to rotavirus therapy. A recent study assessed direct medical costs for the Italian Health System being around 30 million euro per year [19]. Our study reports that DRG costs associated to hospital admissions due to RVGE are lower than the European mean. European data on the expenditure of rotavirus disease showed that hospital admission is a main cost driver, but non-medical costs are not negligible [16]. However, our study does not estimate social costs, indirect costs and family expenditure costs.

In Italy the national vaccination programme does not include rotavirus universal vaccination for newborns despite safe and effective attenuated rotavirus vaccines [Rotarix (GlaxoSmithKline Biologicals s.a.) and RotaTeq (Sanofi Pasteur MSD, SNC)] have been available in Europe since 2006 [20-22]. In view of the lack of interference with co-administered childhood vaccines both could be included within the existing regional and national immunization programmes. Hence Italian public health authorities should consider the opportunity of offering these vaccines to all newborns within 6 months of age [23,24]. Several potential strategies to provide rotavirus vaccination are possible. Firstly, an offer concerning children at greater risk of severe illness (i.e.: low birth weight and preterm infants) can be considered for budgetary reasons. However according to a study conducted in the United States even if those risk factors significantly predicted infants at high risk for hospitalization with viral AGE in the first year of life were neither sufficiently sensitive nor specific to be used to create a focused rotavirus immunization policy [25]. Secondly vaccination could be offered free of charge or in co-payment. As other vaccines routinely offered in Italy Rotavirus vaccine can be administered in primary care settings throughout Departments of prevention and/or paediatricians ambulatories. This choice requires a careful evaluation of local health systems organisation, expenditures and expected coverage have to be considered. The decision on which strategy put into practice should be taken by national and regional authorities according to scientific evidence, international recommendations, local epidemiology and primary care organisations. In addition it needs to be preceded by a cost-effectiveness analysis assessing sustainability.

The strengths of the study include a large study population and the provision of epidemiological information on severe RVGE disease in the area which constitutes a valuable baseline for the future assessment of rotavirus vaccine impact.

There are several limitations of our study. Firstly using administrative data could lead to an underestimation of the RVGE incidence because while HDD are characterized by a high specificity, sensitivity could be lower. However in Italy, as in many European countries, rotavirus is not a notifiable disease and estimates are based mainly on hospital discharge data. The HDD is characterized by high specificity and low sensitivity [14] because of a large number of discharges codified by non-specific codes. The absence of an etiologic diagnosis could be related to budgetary reasons, to not enough sensitive laboratory tests as well as to empirical treatment [2,17]. Furthermore, the hospitalisation rate could be underestimated because secondary diagnosis of rotavirus gastroenteritis were not taken into account. Some authors demonstrated that the incidence of RVGE admissions grows consistently considering both diagnosis but it is feasible that the second diagnosis have a nosocomial origin [12,13].

## Conclusions

RVGE represents an important cause of paediatric hospitalisation in the Veneto Region, though the impact of RVGE admissions is underestimated, primarily due to a low sensitivity of hospital discharges and an active surveillance system should be implemented. Our data reaffirm the burden of rotavirus hospitalisations in children aged ≤5 years and the potential health benefits of the vaccination introduction that should be offered to all newborns within 6 months of age.

## Competing interests

The authors declare that they have no competing interests.

## Authors' contributions

MS and VB have participated in conceive the study, have made substantial contribution to analysis and interpretation of the data, to revision and final approval of the manuscript. AG, TB, MCB, CB have contributed to interpretation of data and have been involved in drafting the paper. GC and FP have contributed to the acquisition and analysis of data. All authors have read and approved the final manuscript.

## Pre-publication history

The pre-publication history for this paper can be accessed here:

http://www.biomedcentral.com/1471-2458/10/636/prepub
